# Developmental Inhibitory Changes in the Primary Somatosensory Cortex of the Stargazer Mouse Model of Absence Epilepsy

**DOI:** 10.3390/biom13010186

**Published:** 2023-01-16

**Authors:** Muhammad Hassan, David R. Grattan, Beulah Leitch

**Affiliations:** Department of Anatomy, School of Biomedical Sciences, Brain Health Research Centre, University of Otago, P.O. Box 913, Dunedin 9054, New Zealand

**Keywords:** GABA_A_ receptor, cortico-thalamocortical network, absence epilepsy, absence seizures, primary somatosensory cortex, stargazer mouse, biochemical fractionation, postnatal development

## Abstract

Childhood absence epilepsy seizures arise in the cortico-thalamocortical network due to multiple cellular and molecular mechanisms, which are still under investigation. Understanding the precise mechanisms is imperative given that treatment fails in ~30% of patients while adverse neurological sequelae remain common. Impaired GABAergic neurotransmission is commonly reported in research models investigating these mechanisms. Recently, we reported a region-specific reduction in the whole-tissue and synaptic GABA_A_ receptor (GABA_A_R) α1 subunit and an increase in whole-tissue GAD65 in the primary somatosensory cortex (SoCx) of the adult epileptic stargazer mouse compared with its non-epileptic (NE) littermate. The current study investigated whether these changes occurred prior to the onset of seizures on postnatal days (PN) 17–18, suggesting a causative role. Synaptic and cytosolic fractions were biochemically isolated from primary SoCx lysates followed by semiquantitative Western blot analyses for GABA_A_R α1 and GAD65. We found no significant changes in synaptic GABA_A_R α1 and cytosolic GAD65 in the primary SoCx of the stargazer mice at the critical developmental stages of PN 7–9, 13–15, and 17–18. This indicates that altered levels of GABA_A_R α1 and GAD65 in adult mice do not directly contribute to the initial onset of absence seizures but are a later consequence of seizure activity.

## 1. Introduction

A developing perinatal brain is highly vulnerable to genetic, neurological, mental, and behavioral disorders due to the immense rapid structural and functional changes at critical stages [1,2,3,4,5,6,7]. Evidence indicates that this developing brain is also more susceptible to seizures than that of an adult [8,9,10]. Indeed, childhood absence epilepsy (CAE) develops early on during childhood, with 3–8 years as the peak age of onset. CAE is a genetic generalized epileptic syndrome, characterized by absence seizures clinically detected as 2.5–4 Hz spike–wave discharges (SWDs). These seizures are believed to arise in the cortico-thalamocortical (CTC) network due to complex underlying mechanisms. The network comprises the cortex and ventroposterior (VP) relay thalamus reciprocally connected via excitatory projections and modulated via inhibitory interneurons in the cortex and reticular thalamic nucleus (RTN) through feedforward and feedback inhibition. Normal development ensures the important excitatory/inhibitory (E/I) balance within the network, disruption of which predisposes individuals towards the development of seizures.

The rapid development of the human brain continues during the first few postnatal years [11,12,13,14], during which thalamocortical relay activity is crucial for the development of cortical networks [15]. The association cortical areas are the fastest growing regions during this period, making them the most vulnerable as well [16,17]. The complexity of cortical neurons rapidly increases, reaching a peak at 2–4 years [18]. Given the time period of absence seizures onset in CAE, an inevitable relationship may exist between an abnormal perinatal development of the CTC network and the pathogenesis of absence seizures [19,20].

Interestingly, the common rodent models for absence seizures used to study human absence epilepsy also suggest a causative correlation with cellular and molecular changes in postnatal brain development, given the onset of seizures in later postnatal stages [21]. In the stargazer mouse model for absence epilepsy used in the current study, seizures were detected at PN 17–18 [22], which, in terms of the brain development time course in mice, is roughly equivalent to the time of onset in humans [8,22,23]. The primary genetic defect of the stargazin gene in the mouse leads to the reduced expression and function of the α-amino-3-hydroxy-5-methyl-4-isoxazolepropionic acid (AMPA) receptors in various brain regions with evidence of downstream changes in GABAergic neurotransmission [24,25,26,27,28,29,30,31,32]. We have previously reported that such loss of AMPA receptors occurs both in post-seizure adults and young postnatal stargazers prior to the age of seizure onset [33,34,35]. Recently, we reported a reduced expression of the synaptic GABA_A_ receptor (GABA_A_R) α1 subunit [36], an increase in global whole-tissue GABA neurotransmitter content but reduced GABA levels in individual inhibitory synaptic terminals [37], and an increase in global whole-tissue GAD65 but not GAD67 in adult stargazer primary SoCx, which was suggestive of altered inhibition [38].

Given that we have found no change in the number of GABA-positive neurons and immunoreactive profiles [39], one implication of the higher tissue levels of GABA despite lower levels within terminals is a persistent increase in GABA in the extracellular space. Such an increase has previously been linked to absence seizure generation [40,41]. The inference of increased extracellular GABA is also substantiated by the increased GAD65, but not GAD67, in adult stargazer primary SoCx [38]. GAD65 is an enzyme with a preferential localization in the axon terminals for on-demand GABA synthesis and release [42]. Its activity is crucial during intense synaptic activity [43]. Since GADs are the rate-limiting enzyme in the synthesis of GABA [44], there appears to be a good correlation between the demand for GABA and GAD65 levels in the neuronal tissue. Hence, GAD65 is an acceptable representative enzyme for GABA levels in stargazer primary SoCx. Given that it is unknown whether the altered expressions of synaptic GABA_A_R α1, the GABA neurotransmitter, and GAD65 in adult stargazer primary SoCx are a potential cause or a downstream consequence of absence seizures, the current study investigated changes in synaptic GABA_A_R α1 and GAD65 at critical postnatal stages prior to the onset of seizures.

Most studies describing developmental changes in rodent GABAergic neurotransmission have been conducted in rats. GABA and GABA_A_R’s developmental patterns are crucial for the physiological regulation of the rapidly proliferating and migrating neural and glial progenitors [45,46]. GABAergic neurotransmission during early rodent perinatal development is excitatory and crucial for normal neural development [47], with the switch to inhibitory neurotransmission in the CTC network occurring after the first postnatal week [47]. The GABA neurotransmitter is present in the neural system perinatally [45], with its synthesis being dependent on the GAD65 and GAD67 enzymes. GAD67, which is present during birth, achieves adult expression by PN 13, while GAD65 shows a prolonged increase from PN 6 into adulthood [48,49]. Any disruptions in GABAergic neurotransmission during these stages adversely affect normal development. For instance, the GAD65 knock-out mouse model, which displays a high susceptibility to seizures, shows normal cerebral GABA content at birth attributed to normal GAD67, but the GABA levels do not increase significantly after birth, increasing the brain’s susceptibility to seizures. This is unlike normal wild-type mice in which GABA content increases significantly and rapidly as the brain develops [50,51].

Similarly, GABA_A_Rs, which are expressed during embryonic stages [52,53,54], show prolonged postnatal changes in subunit expression, which continues well into adulthood. For instance, GABA_A_R α1 shows a steady increase in expression into the adult stages, while α2 and α3, which are abundant in the rodent neonatal brain, show a steady parallel decrease in the rat brain [49,55,56,57,58] due to a developmental switch. A similar developmental switch from α2 and α3 to α1 has also been reported in the mouse brain [59,60]. The type of α subunit expressed in a GABA_A_R affects its physiological properties. Naturally, any disruption in the normal composition of GABA_A_Rs can disrupt the physiological E/I balance. Interestingly, a recent publication reported impairment of the brain’s inhibitory networks and development of seizures due to GABA_A_R α1 loss of function in a juvenile zebrafish model [61].

**Figure 1:**
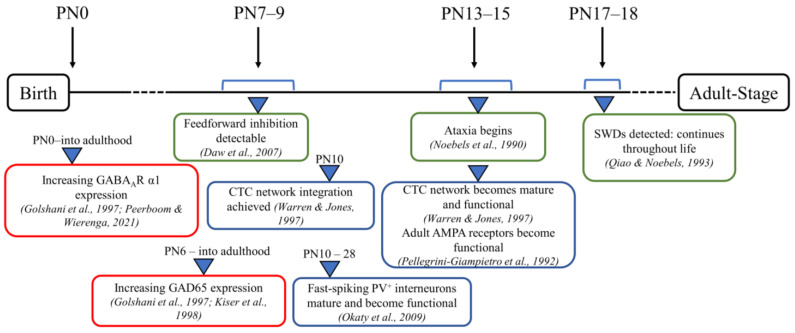
Timeline of critical postnatal development time points in stargazer mice. Schematic representation of developmental timepoints related to the CTC network with green-bordered boxes showing phenotypic changes specifically in stargazer mice, blue-bordered boxes showing CTC network maturation, and red-bordered boxes briefly pointing out developmental patterns of GABA_A_R α1 subunit and GAD65 [22,47,48,49,62,63,64,65,66].

It is conceivable that the GABAergic changes observed in adult stargazer primary SoCx could have an onset at critical time points during the development of the CTC network prior to the onset of seizures that could contribute to seizure generation. Hence, the current study was conducted to test our hypothesis that modified expression of the stargazin gene leads to downstream changes in synaptic GABA_A_R subunit α1 and GAD65 that take effect during the developmental stages of a stargazer’s neuronal development. To assess this, we selected three developmental stages in juveniles (Figure 1). At PN 7–9, feed-forward inhibition is detectable, while GABAergic transmission switches from excitatory to inhibitory, and the GABA_A_R subunit switch to α1 has occurred [47,58,62]. At PN 13–15, adult AMPA receptors become functionally active, and the CTC network is operational [63,64]. At PN 17–18, seizures are first detected in stargazers [65]. Biochemical fractionation was first utilized to isolate the synaptic and cytosolic subcellular components of the primary SoCx from NE control littermates and stargazers at the three juvenile stages followed by probing for synaptic GABA_A_R subunit α1 and cytosolic GAD65.

## 2. Materials and Methods

### 2.1. Animals

Male epileptic (E) stargazers (stg/stg) and their male non-epileptic (NE: heterozygous [Het, +/stg] and wild-type [WT, +/+]) control littermates were used in all experiments. These offspring were obtained from stargazer (*B6C3Fe a/a-Cacng2^stg^/J*) breeding stocks obtained from the Jackson Laboratory (Bar Harbor, ME, USA). All animals used in the study were raised at the University of Otago’s Animal Resource Unit. The mice had ad libitum access to food and were housed in well-ventilated cages with optimum environmental conditions (12 h light/dark cycle, ~21 °C temperature, and ~50% humidity). All animal procedures were carried out according to the University of Otago Animal Ethics Committee approved protocols (32/17). Animals selected were juvenile pups from 3 age groups, PN 7–9, 13–15, and 17–18, marking 3 stages of neuronal development in mice. After obtaining ear-notches post-sacrifice, genotypes were confirmed with PCR-amplified DNA using the primer sequences oIMR9601 (TAC TTC ATC CGC CAT CCT TC), oIMR9602 (TGG CTT TCA CTG TCT GTT GC), and oIMR8983 (GAG CAA GCA GGT TTC AGG C). A total of 82 animals (41 NE control littermates and 41 epileptic stargazers) were used for the current study with *n* numbers representing the sample numbers used. Subcellular fractions from PN 7–9 brains were pooled based on the same age and genotype to increase the protein content. The GAD65 group *n* numbers are one less for PN 13–15 and 17–18 because of a lack of enough protein in the excluded samples.

### 2.2. Biochemical Fractionation

Juvenile mice were weaned before being brought in for decapitation, followed immediately by brain extraction and snap-freezing on dry ice. The brains were coronally sectioned in a rostral-to-caudal direction at 200 μm thickness using a vibratome (Leica VT1200, Nussloch, Germany). Sections obtained were thaw-mounted on glass slides before micro-dissecting the full depth of the primary SoCx region. Tissue samples were homogenized in a fractionation buffer (320 mM sucrose, 10 mM tris-base, and 1 mM EDTA; pH 7.37) and supplemented with 1% PMSF (phenylmethyl sulfonyl fluoride) and a 1% protease inhibitor (P8340, Sigma-Aldrich, St. Louis, MO, USA), using clean sterilized plastic pestles coupled with ultrasonication. The subcellular components (total lysate, cytosol, and extra-synaptic and synaptic fractions) were obtained by taking each of the homogenized tissue samples through a previously established multi-step centrifugation fractionation technique [34,67,68]. Centrifugation at 1000× *g* for 10 min pelleted unhomogenized debris and nuclei. The supernatant (total lysate) was centrifuged for 15 min at 10,000× *g,* pelleting the cell membrane and yielding the cytosol fraction as a supernatant. The cell membrane pellet was resuspended in an ice-cold homogenization buffer (50 mM Tris, 2 nM EDTA, and 3% SDS; pH 6.8), supplemented with 0.5% Triton X-100, incubated for 40 min on ice, and centrifuged at 16,000× *g* for 30 min at 4 °C. This yielded a pelleted synaptic fraction due to its insolubility in Triton X-100 with a supernatant containing the extra-synaptic fraction. The synaptic fraction was resuspended in the homogenization buffer, while acetone was added to the extra-synaptic fraction and incubated overnight at −20 °C followed by centrifugation at 3000× *g* for 15 min. The resulting extra-synaptic pellet was resuspended in the homogenization buffer.

### 2.3. Western Blotting of Subcellular Fractions

The protein content of each sample was assessed using a detergent-compatible protein assay (DC Protein Assay, 500, 0116, Bio-Rad, Hercules, CA, USA). Western blot analyses were carried out as per previously established protocols [33,34]. In brief, the proteins were separated in 8.5% resolving gels using SDS-PAGE. After transferring them onto nitrocellulose membranes, the protein expression was probed using relevant antibodies. The synaptic component was probed for the principal protein of interest GABA_A_R α1 (1:500; AGA-001, Alomone, Jerusalem, Israel) and marker proteins with PanC (1:1000; 4068P, Cell Signaling Technology, Danver, MA, USA) for normalization and analyses, and PSD-95 (1:1000; 124011, Synaptic Systems, Goettingen, Germany) and β-actin (1:1000; ab8226, Abcam, Cambridge, UK) were used as additional controls. The cytosol component was probed for the principal protein of interest GAD65 (1:1000, ab26113, Abcam, Cambridge, UK) and the marker proteins. Although this study was focused on the synaptic and cytosol components, all subcellular components were taken through Western blot runs to establish the specificities of the fractions. Protein standard (Novex Sharp Pre-stained Protein Standard, Life Technologies, LC5800) was added to each Western blot run for molecular weight reference. Protein expression was detected using secondary antibodies IRDye 800CW (1:10,000; 926-3221, LI-COR Biosciences, Lincoln, NE, USA) and IRDye 680 (1:10,000; 926-32210, LI-COR Biosciences, Lincoln, USA). An Odyssey infrared imager (Li-COR Biosciences, Lincoln, NE, USA) was used to detect the immunofluorescence, while Odyssey v3.1 (Li-COR Biosciences, Lincoln, NE, USA) software was used to estimate the intensities of each band. The intensities of the protein of interest were normalized against PanC followed by a statistical analysis comparing stargazers with their NE control littermates.

### 2.4. Statistical Tests

A non-parametric Kruskal–Wallis one-way ANOVA test followed by a post hoc Dunn’s multiple comparison test for pairwise comparison between the groups were used to probe the data for developmental changes across the three age groups in NE control littermates and stargazers. An unpaired non-parametric Mann–Whitney U test was used for the data analysis and comparison between the two independent groups of NE control littermates and stargazers from the same population. The independent variable between the two groups was the existence of a genetic mutation-based phenotype in the stargazers and the lack thereof in the control group. The data obtained from each experiment conducted represent dependent variables. The statistical comparison between the NE control littermates and stargazers was conducted in GraphPad Prism (GraphPad Software Inc., San Diego, CA, USA). Significance was defined as *p* < 0.05 as *; *p* < 0.01 as **; and *p* < 0.001 as ***. The data are presented as means ± standard error of the mean (SEM).

## 3. Results

### 3.1. Biochemical Fractionation Technique Verification

**Figure 2:**
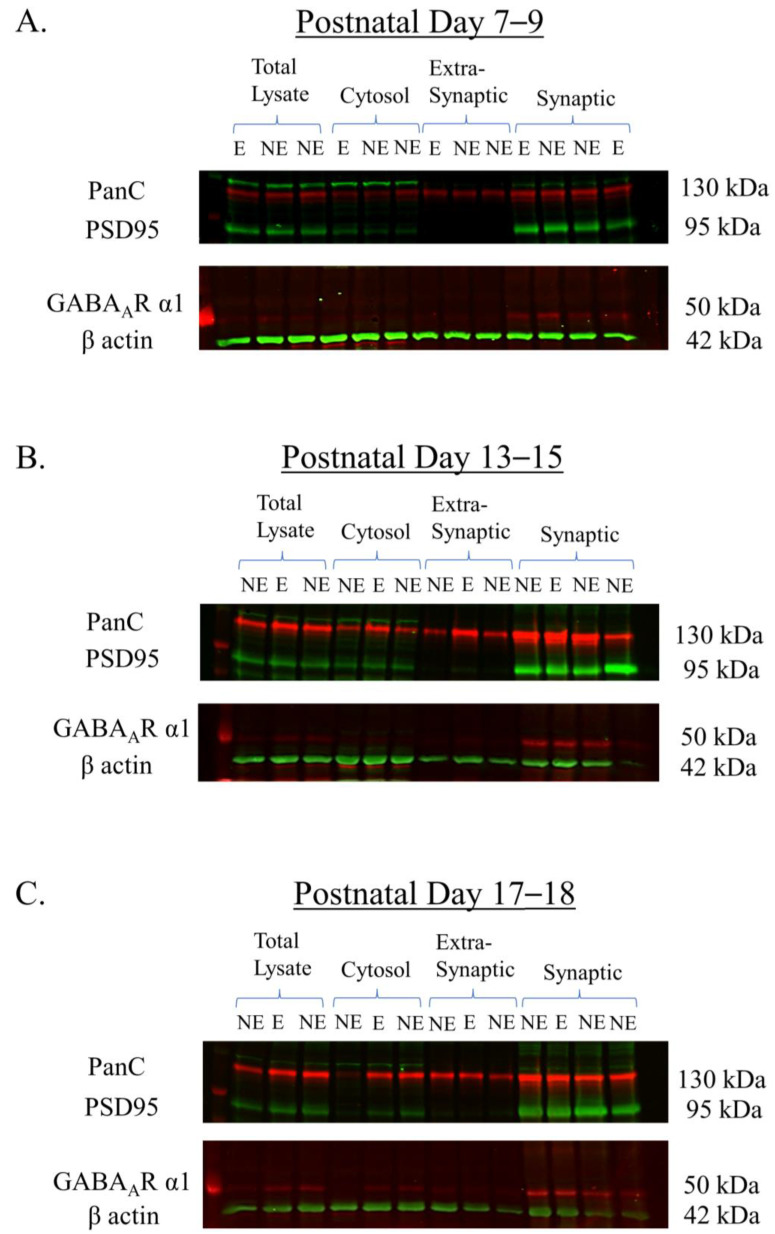
Representative Western blots showing the expression of GABA_A_R α1 in subcellular components (total lysate, cytosol, and extra-synaptic and synaptic). Subcellular fractions were derived from the primary SoCx of NE control littermates and epileptic (E) stargazers at PN 7–9 (**A**), 13–15 (**B**), and 17–18 (**C**). Note that the synaptic fraction shows intense signals for PSD95 while the extra-synaptic shows the complete absence of PSD95. PanC shows expression in all subcellular fractions. GABA_A_R α1 shows intense enrichment in synaptic fractions.

A pilot Western blotting study was first conducted to validate the purity of the subcellular fractions derived from the primary SoCx using biochemical fractionation in the 3 juvenile age groups PN 7–9, 13–15, and 17–18. The proteins on the Western blots were probed using antibodies for the PanC, PSD95, and β-actin marker proteins, and GABA_A_R α1. Figure 2 shows the representative Western blots with immuno-positive fluorescence bands detected at the manufacturers’ specified molecular weights as follows: PanC at ~130 kDa, PSD95 at 95 kDa, GABA_A_R α1 at ~50 kDa, and β-actin at 42 kDa. PanC, a transmembrane glycoprotein, was detected in all fractions [69] and was used as the normalization protein because its expression pattern is not dependent on the stargazin gene and its expression remains unaltered between NE control littermates and stargazers, as previously reported in [68,70]. PSD95 is a component of the post-synaptic density [71,72], and, as expected, showed intense enrichment in the synaptic fraction with no bands seen in the extra-synaptic fraction, demonstrating the successful isolation of the synaptic subcellular component due to its insolubility in the non-ionic detergent Triton X-100. β-actin was seen in all fractions but was not used for the normalization analysis because of its inconsistent expression in membranous components as well as its association with other membranous proteins [34,70,73]. GABA_A_R α1 displayed enrichment in synaptic fractions, which was expected given its predominant expression at GABAergic synapses. Conversely, extra-synaptic fractions displayed no visible bands. GABA_A_R α1 also showed faint bands in the total lysate and cytosolic fractions, given that this protein is synthesized in the cytoplasm.

### 3.2. Synaptic GABA_A_R α1 Is Unaltered in the Stargazer Primary SoCx

**Figure 3:**
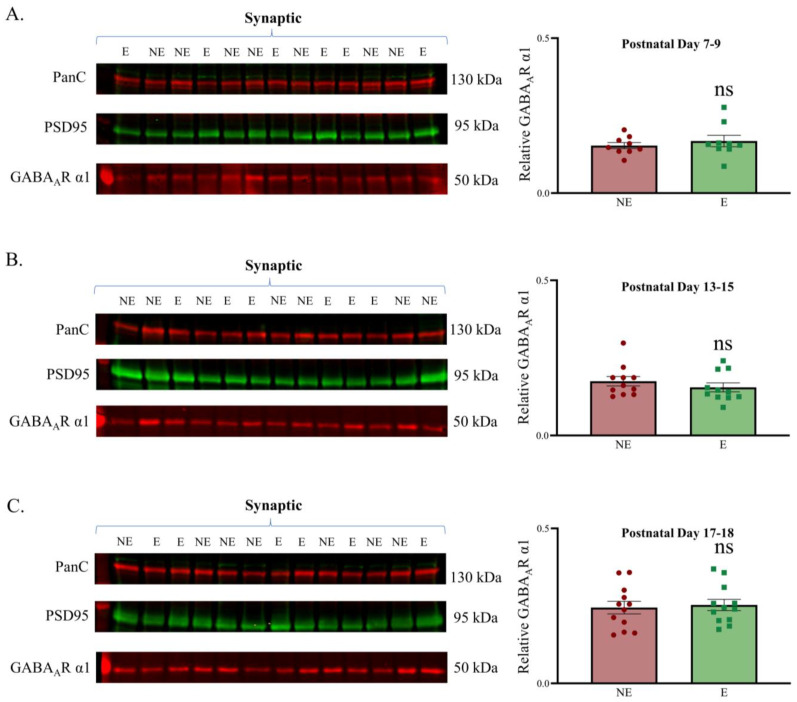
Relative synaptic expression of GABA_A_R α1 in the developing primary SoCx. GABA_A_R α1 remains unchanged in the epileptic (E) stargazer primary SoCx compared with their NE control littermates in all 3 age groups: (**A**) PN 7–9 (NE 0.153 ± 0.009, *n* = 9; E 0.168 ± 0.018, *n* = 9; *p* = 0.546), (**B**) PN 13–15 (NE 0.174 ± 0.015, *n* = 11; E 0.155 ± 0.014, *n* = 11; *p* = 0.235), and (**C**) PN 17–18 (NE 0.245 ± 0.020, *n* = 12; E 0.253 ± 0.018, *n* = 12; *p* = 0.671). The significance threshold was set at 0.05. ‘ns’ is indicative of no significant change found.

Having established the purity of subcellular fractions, we next assessed the levels of synaptic GABA_A_R α1 in the juvenile stargazer primary SoCx compared with their NE counterparts before the onset of seizures by probing Western blots containing synaptic fractions with GABA_A_R α1 antibodies. GABA_A_R α1 showed good enrichment in the synaptic fractions. The analyses demonstrated that synaptic GABA_A_R α1 expression in the stargazer primary SoCx was not significantly different from that in the NE control littermates at each development time point (Figure 3). Representative original Western blots for all subcellular fractions are provided in Supplementary Figure S1 (PN 7–9), Supplementary Figure S2 (PN 13–15), and Supplementary Figure S3 (PN 17–18).

**Figure 4:**
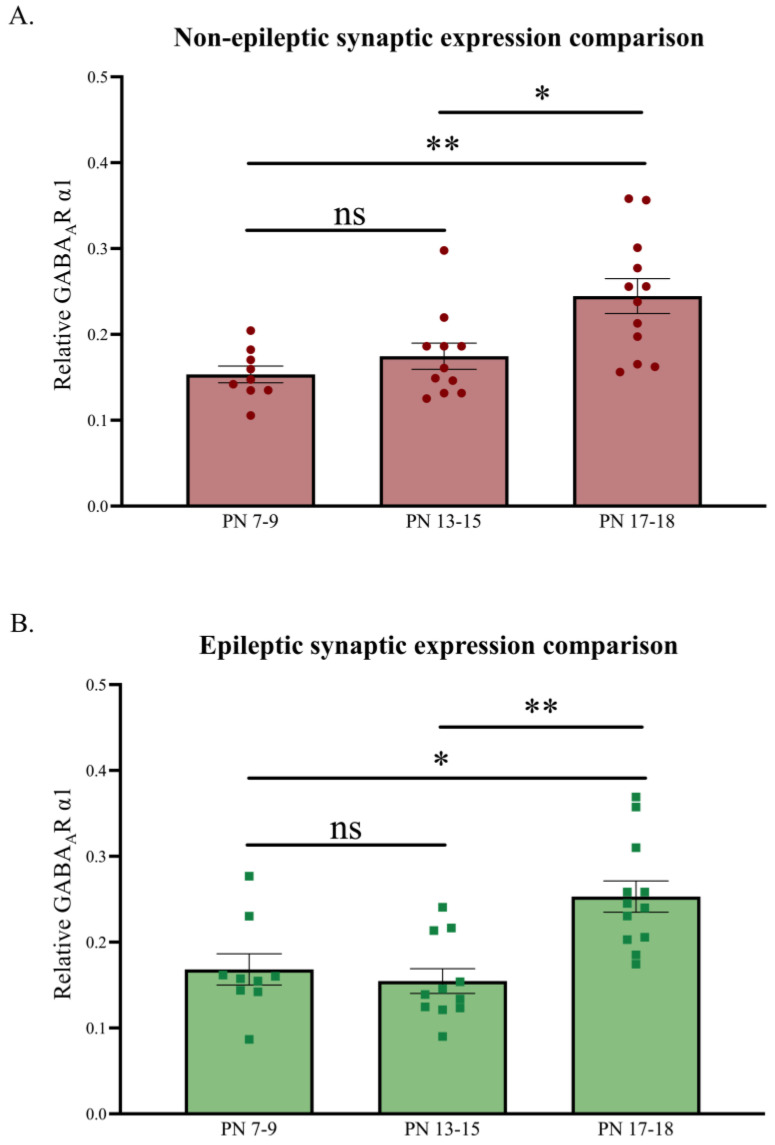
Relative developmental changes in synaptic GABA_A_R α1 expression levels in the primary SoCx. Synaptic GABA_A_R α1 expression levels increase with increasing postnatal age in both NE littermate controls and epileptic stargazers. (**A**) Kruskal–Wallis ANOVA results show a significant increase from PN 7–9 to 17–18 (*H(2)* = 11.49; *p* = 0.0032; **); post hoc Dunn’s multiple comparison test, shown on the graph, reveals a significant increase in synaptic GABA_A_R α1 from PN 7–9 to 17–18 (*p* = 0.031), PN 13–15 to 17–18 (*p* = 0.001), and PN 7–9 to 13–15 (*p* > 0.999). (**B**) Kruskal–Wallis ANOVA results show a significant increase from PN 7–9 to 17–18 (*H(2)* = 13.72; *p* = 0.0032; **); post hoc Dunn’s multiple comparison test, shown on the graph, reveals a significant increase in synaptic GABA_A_R α1 from PN 7–9 to 17–18 (*p* = 0.031), PN 13–15 to 17–18 (*p* = 0.001), and PN 7–9 to 13–15 (*p* > 0.999). The significance threshold was set at 0.05 with * and ** indicating *p* < 0.05 and *p* < 0.01, respectively. ‘ns’ is indicative of no significant change found.

The relative synaptic GABA_A_R α1 levels, normalized against PanC, were also used to analyze developmental changes across the three juvenile age groups for both the NE control littermates and stargazers (Figure 4). As expected, synaptic GABA_A_R α1 revealed a significant increase across the three developmental stages [49,74]. Synaptic GABA_A_R α1 expression showed a ~123% increase in NE control littermates (*n* = 32; *p* = 0.0032) and a ~77% increase in stargazers (*n* = 32; *p =* 0.0032) from PN 7–9 to 17–18, with the increase being notably significant between PN 13–15 and 17–18.

### 3.3. GAD65 Is Unaltered in the Stargazer Primary SoCx

**Figure 5:**
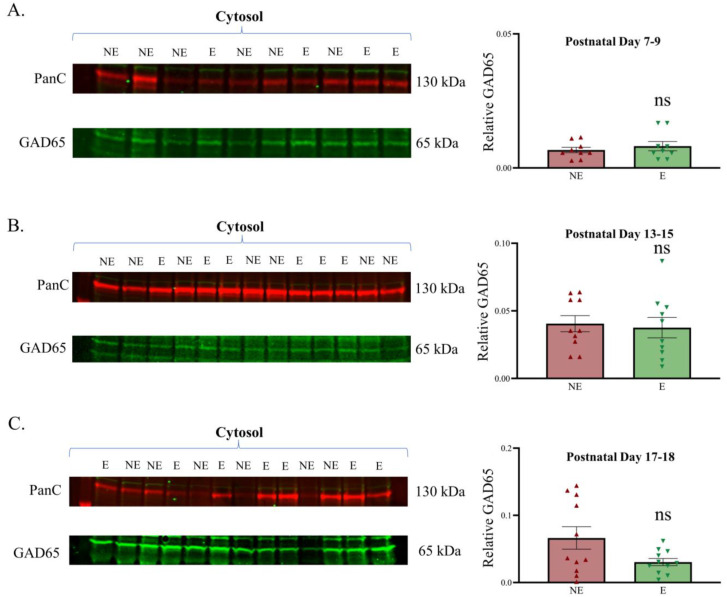
Relative expression of GAD65 in the developing primary SoCx. Western blot analyses of GAD65 reveal a lack of significant change in the epileptic (E) stargazer primary SoCx prior to the onset of seizures compared with NE control littermates. Assessments were conducted in cytosol fraction at PN 7–9, 13–15, and 17–18. (**A**) PN 7–9 (NE 0.007 ± 0.001, *n* = 9; E 0.008 ± 0.002, *n* = 9; *p* = 0.779), (**B**) PN 13–15 (NE 0.040 ± 0.006, *n* = 10; E 0.037 ± 0.007, *n* = 10; *p* = 0.517), and (**C**) PN 17–18 (NE 0.066 ± 0.017, *n* = 11; E 0.030 ± 0.005, *n* = 11; *p* = 0.217). The significance threshold was set at 0.05. ‘ns’ is indicative of no significant change found.

Previously, we reported that the GABA neurotransmitter was significantly increased globally throughout the primary SoCx (as revealed with HPLC whole-tissue analyses) but reduced within each inhibitory terminal (as revealed with immunogold ICC-EM analyses) of adult epileptic stargazers [37]. We further found that GABAergic neuron population density and immunoreactive profile density within the neuropil were not significantly different in the adult stargazer primary SoCx compared with their NE control littermates, which suggested that the likely increase in tissue GABA levels was extracellular rather than a consequence of the increased branching of GABAergic axons within the neuropil during development [39]. The adult stargazer primary SoCx also showed a significant increase in GAD65 expression [38]. Given the correlation between GAD65 levels and changes in the synthesis/release of GABA at presynaptic terminals, GAD65 was assessed during the stargazers’ development using a Western blot analysis of the cytosol fractions at PN 7–9, 13–15, and 17–18. Bands were detected at the expected molecular weight of 65 kDa. Analyses after normalization against PanC showed no difference between GAD65 expression levels in the NE control littermate and stargazer primary SoCx (Figure 5). Representative original Western blots for all subcellular fractions are provided in Supplementary Figure S1 (PN 7–9), Supplementary Figure S2 (PN 13–15), and Supplementary Figure S3 (PN 17–18).

**Figure 6:**
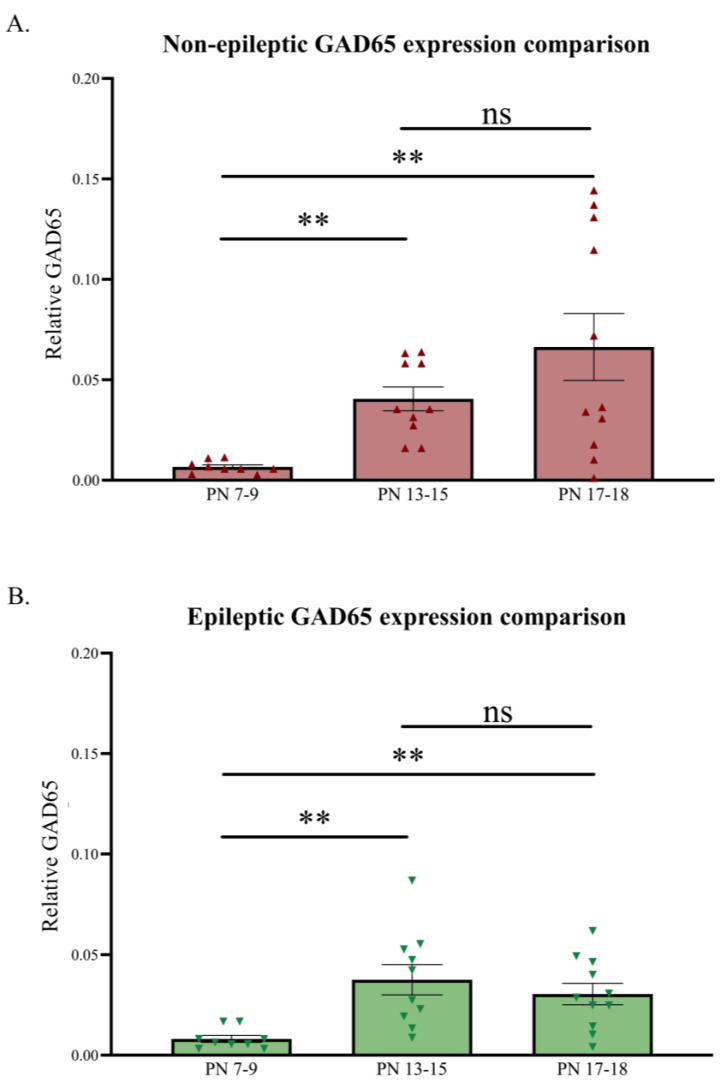
Relative developmental changes in GAD65 levels in the primary SoCx. GAD65 expression levels increase with increasing postnatal age in the NE control littermates and epileptic (E) stargazers, although the stargazers show a sudden dip at PN 17–18. (**A**) Kruskal–Wallis ANOVA results show a significant increase from PN 7–9 to 17–18 (*H(2)* = 14.36; *p* = 0.0008; **); post hoc Dunn’s multiple comparison test results, shown on the graph, reveal a significant increase in GAD65 from PN 7–9 to 17–18 (*p* = 0.002), PN 13–15 to 17–18 (*p* > 0.999), and PN 7–9 to 13–15 (*p* = 0.005). (**B**) Kruskal–Wallis ANOVA results show a significant increase from PN 7–9 to 17–18 (*H(2)* = 13.20; *p* = 0.001; **); post hoc Dunn’s multiple comparison test reveals a significant increase in GAD65 from PN 7–9 to 17–18 (*p* = 0.009), PN 13–15 to 17–18 (*p* > 0.999), and PN 7–9 to 13–15 (*p* = 0.002). The significance threshold was set at 0.05, with ** indicating *p* < 0.01. ‘ns’ is indicative of no significant change found.

Changes in the relative cytosolic GAD65 levels, normalized against PanC, across the three developmental age groups were also analyzed for both NE control littermates and epileptic stargazers (Figure 6). As expected, GAD65 revealed a significant increase across the three developmental stages [49,75]. Expression patterns showed a ~220% increase in NE control littermates (*n* = 30; *p* = 0.0008) and a ~175% increase in stargazers (*n* = 30; *p* = 0.001) from PN 7–9 to 17–18, with the relatively significant increase occurring between PN 7–9 and 13–15.

## 4. Discussion

The current study investigated whether the altered expressions of the GABA_A_R α1 subunit and GAD65, seen in adult stargazer primary SoCx, were established prior to the onset of seizures (at PN18) by analyzing their expressions at 3 development stages between PN 7–18. Both the NE control littermates and stargazers showed significant increases in the expressions of synaptic GABA_A_R α1 and cytosolic GAD65 in the primary SoCx during development from PN7–9 to PN17–18. However, no statistically significant difference was found in synaptic GABA_A_R α1 and cytosolic GAD65 levels in epileptic stargazers compared with their age-matched NE littermate controls, at any of the three developmental stages. These data suggest that aberrant GABAergic expression found in adult stargazer primary SoCx does not occur prior to the onset of seizures, and hence may not contribute directly to the pathogenesis of seizures but perhaps is a secondary compensatory change that could either play a role in limiting seizure generation or alternatively potentially contribute to the maintenance of seizures.

The first three postnatal weeks (in rodent models) are the most important in terms of the morphological and functional maturation of neurons and synaptogenesis. Hence, we first determined the developmental patterns for synaptic GABA_A_R α1 and cytosolic GAD65 during PN 7–9, 13–15, and 17–18. Our results showed a steady overall increase in both GABA_A_R α1 and GAD65, which is consistent with previous reports [76,77]. A similar expression pattern for GABA_A_R α1 has been reported in rats where α1 is low at birth but shows a gradual increase thereafter up until about three weeks of age when the expression is widespread in most brain areas, including the neocortex [55,58]. Fritschy et al. (1994) also reported the region-specific maturation of α1 with, for instance, the primary SoCx preceding association areas by several days, which is perhaps one explanation for why the increase in expression levels was slower between PN 7–15 (Figure 4). A similar pattern of change in the expression of postnatal GABA_A_R α1 has also been reported in another mouse model, wherein the gene expressions of GABA_A_R subunits and GADs in the primary SoCx were studied using in situ hybridization histochemistry [49]. In the current study, GAD65 also showed a marked developmental increase from PN 7–9, but the increase became comparatively slower from PN 13–18, which was likely due to reaching adult levels at around the 3 weeks mark, as previously reported in [48]. Kiser et al. (1998) reported that in rats, there was a minimal presence of GAD65 prior to PN 6 but a rapid increase thereafter. When comparing the developmental expression pattern of GAD65 between stargazers and NE control littermates, a relative decrease in GAD65 at PN 17–18 in the stargazer primary SoCx was seen (Figure 6). This coincides with the stage at which fast-spiking properties of parvalbumin-positive (PV^+^) interneurons become active and absence seizures SWDs are detected [78,79,80]. It is possible that the dysfunctional engagement of fast-spiking PV^+^ feed-forward inhibitory interneurons in stargazer primary SoCx results in a transient reduction in GAD65.

Multiple studies implicate impaired GABAergic neurotransmission in the CTC network pathognomonic of absence seizures [81,82,83,84,85,86]. Similarly, we reported altered GABAergic neurotransmission in the adult stargazer primary SoCx in the form of reduced synaptic GABA_A_R α1 expression and a potential increase in ambient GABA levels supplemented by the findings of increased GAD65 [36,38]. Such disruptions could contribute to absence seizure generation in the CTC network [82]. Thus far, many studies have reported impaired GABAergic neurotransmission in the adult CTC network, but very few have investigated the developing CTC network in epilepsy models prior to the onset of seizures. One such study conducted at PN 25 in the Genetic Absence Epilepsy Rats from Strasbourg (GAERS), prior to the age when absence seizures are fully established in this rodent model, reported an increase in the density of GABAergic interneurons in the primary SoCx and RTN and an increased GABAergic innervation of relay neurons in the ventroposterior (VP) thalamus [41]. A two-fold increase in tonic currents in the VP relay thalamus at PN 17 has also been reported in these GAERS [40]. Conversely, in the stargazer mouse, direct impairment of GABA-mediated neurotransmission, in the form of altered GABA_A_R subunit expression in the primary SoCx (the current study) and VP relay thalamus [30,31,32], and altered tonic inhibition in the VP relay thalamus [40,87], occurs after seizure onset and is secondary to dysfunctional feedforward inhibition [33,34,82,88,89].

In stargazers, the onset of seizures is related to dysfunctional feedforward inhibitory microcircuits within the CTC network [90,91] as a result of AMPA receptor deficits in the GABAergic fast-spiking PV^+^ interneurons [35]. Chemogenetic inactivation of these feedforward inhibitory interneurons using Designer Receptor Exclusively Activated by Designer Drug (DREADD) technology induced absence seizures in normal mice [89], whereas activation of the same neurons during absence seizures reduced them [90]. The genetic defect in the stargazers results in the loss of stargazin, a transmembrane AMPA receptor regulatory protein (TARP) that regulates synaptic AMPA receptor expression. Stargazin is the only TARP expressed in PV^+^ interneurons in the CTC network, and it is expressed in the perinatal stages [92]. While evidence shows AMPA receptor subunit reduction in stargazers as far back as PN 13–15, a stage at which mature calcium-permeable AMPA receptors are expressed [63], the onset of seizures themselves occurs at PN 17–18. Given that PV^+^ interneurons begin engaging their fast-spiking properties during this stage [78,79,80], a correlation between dysfunctional fast-spiking PV^+^ interneuron-mediated phasic inhibition and the initiation of seizures is indicated. Our findings indicate that it is past this point that altered GABAergic neurotransmission (i.e., altered GABA receptor and neurotransmitter expression) occurs, as the neural system continues to functionally and structurally mature while affected by SWDs. This could be a compensatory response to overcome overexcitations in the cortical circuitry or downstream effects from overexcitations that may further potentiate or maintain SWDs.

Structural and functional plasticity in neural circuits regulates and maintains the homeostatic E/I balance [93], failure of which results in neurological disorders including epilepsy [94,95,96]. In stargazers, chronic loss of key proteins (the stargazin and AMPA receptor subunits) and the consequential dysfunctional inhibitory CTC microcircuits and resultant downstream changes permit the generation and maintenance of absence seizures. Given that pyramidal neurons comprise around 80% of the cortical neurons, it is possible that hyperexcitations in the pyramidal neurons lead to intense firing in other GABAergic interneurons causing a release of GABA from multiple sites in adult stargazer primary SoCx [97], which may explain the increased levels of GAD65 and extracellular GABA. Increased extracellular GABA-mediated enhanced tonic inhibition in thalamocortical relay neurons due to compromised GABA re-uptake by GABA transporter-1 (GAT-1) has been reported by Cope et al. (2009) in GAERS, stargazers, and lethargic genetic models for absence epilepsy [40]. Cope et al. (2009) postulated that this could be the mechanism driving absence seizures; however, these changes were reported in adult animals with fully established seizures and not in juveniles. Whether a similar mechanism operates in the primary SoCx of stargazer mice remains to be investigated. Future studies should be conducted to confirm if this is indeed the case. The microdialysis method, for instance, can be used to measure the levels of GABA before and after the onset of seizures in stargazers, while intracellular electrophysiological recordings can give insights into the activity of cortical neurons. Although an increase in extracellular GABA in adult stargazer primary SoCx might be expected to suppress seizures, it could in fact lead to their enhancement via disinhibitory circuits or enhanced tonic-inhibition-mediated T-type calcium channel burst firing [40,87,98,99]. The fact that the use of GABAergic inhibition-enhancing drugs aggravates absence seizures supports the concept that secondary GABAergic changes past the age of seizure onset may have pro-seizure effects rather than the opposite [100,101,102,103,104].

## 5. Conclusions

The current study has clearly shown that changes in synaptic GABA_A_R α1 and cytosolic GAD65 expression do not occur prior to absence seizure onset in the stargazer primary SoCx. This would indicate that changes in synaptic GABA_A_R α1 and GAD65 are not responsible for initiating seizures but are secondary compensatory changes. However, developmental changes in neuronal networks followed by secondary compensatory changes and the pathogenesis of absence seizures are clearly inter-dependent [105]. Therefore, these post-seizure compensatory changes could clearly be involved in the ongoing maintenance of seizures, even if they are not causative of their initiation. The timeline for such changes should be taken into account when designing targeted therapeutics, as the outcome of treatment could be affected. It is important to note that our current study assessed changes in all layers of the primary SoCx collectively, while pre-seizure layer-specific changes may be present that could be important in causing absence seizures. Recent evidence indicates the presence of cortical layer cell-specific changes in stargazer primary SoCx [106]. Future investigations could use techniques, such as laser microdissection, to procure layer-specific tissue, coupled with quantitative protein expression analyses (for instance, Western blotting or liquid chromatography/mass spectrometry) to identify any layer-specific changes that may have been masked when the whole-tissue primary SoCx were assessed in our study.

## Data Availability

The data are available upon reasonable request.

## Supplementary Materials

The following supporting information can be downloaded at https://www.mdpi.com/article/10.3390/biom13010186/s1. Figure S1: representative Western blots for subcellular fractions at PN 7–9, Figure S2: representative Western blots for subcellular fractions at PN 13–15, and Figure S3: representative Western blots for subcellular fractions at PN 17–18.
