# Supplementary material for: Developmental Inhibitory Changes in the Primary Somatosensory Cortex of the Stargazer Mouse Model of Absence Epilepsy
Source: Biomolecules. 2023 Jan 16;13(1):186. doi: 10.3390/biom13010186 (PMC9856073; doi:10.3390/biom13010186)
Supplement: Supplementary file 1 [file biomolecules-13-00186-s001.zip › biomolecules-2137645-supplementary.pdf]

biomolecules

# Postnatal Day 7-9

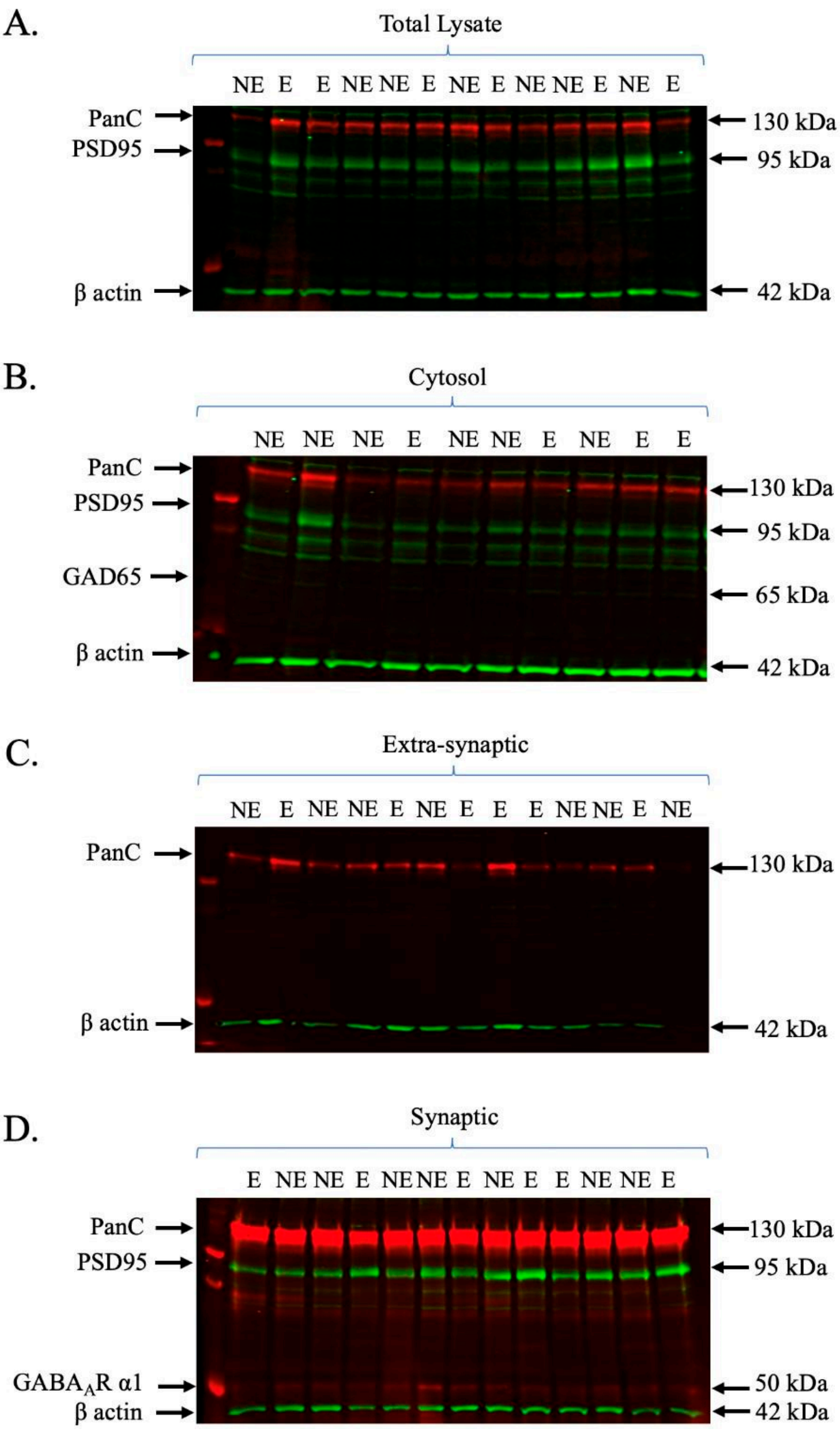


**Figure S1.** Representative western blots for subcellular fractions (total lysate, cytosol, extra-synaptic and synaptic) from primary somatosensory cortex of epileptic stargazers and non-epileptic control littermates at postnatal days 7-9. (NE: Non-epileptic controls; E: epileptic stargazers).

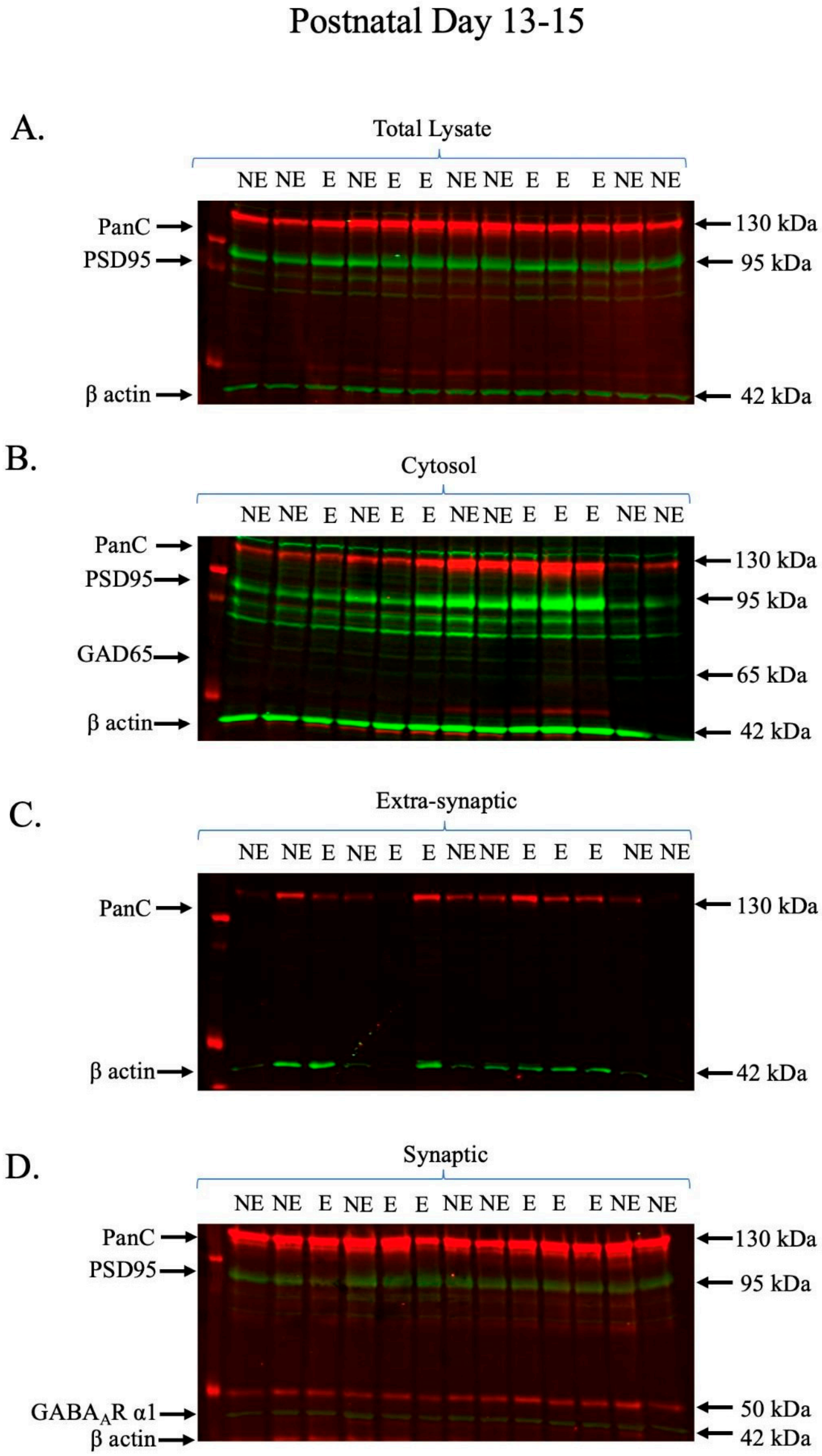


**Figure S2.** Representative western blots for subcellular fractions (total lysate, cytosol, extra-synaptic and synaptic) from primary somatosensory cortex of epileptic stargazers and non-epileptic control littermates at postnatal days 13-15. (NE: Non-epileptic controls; E: epileptic stargazers).

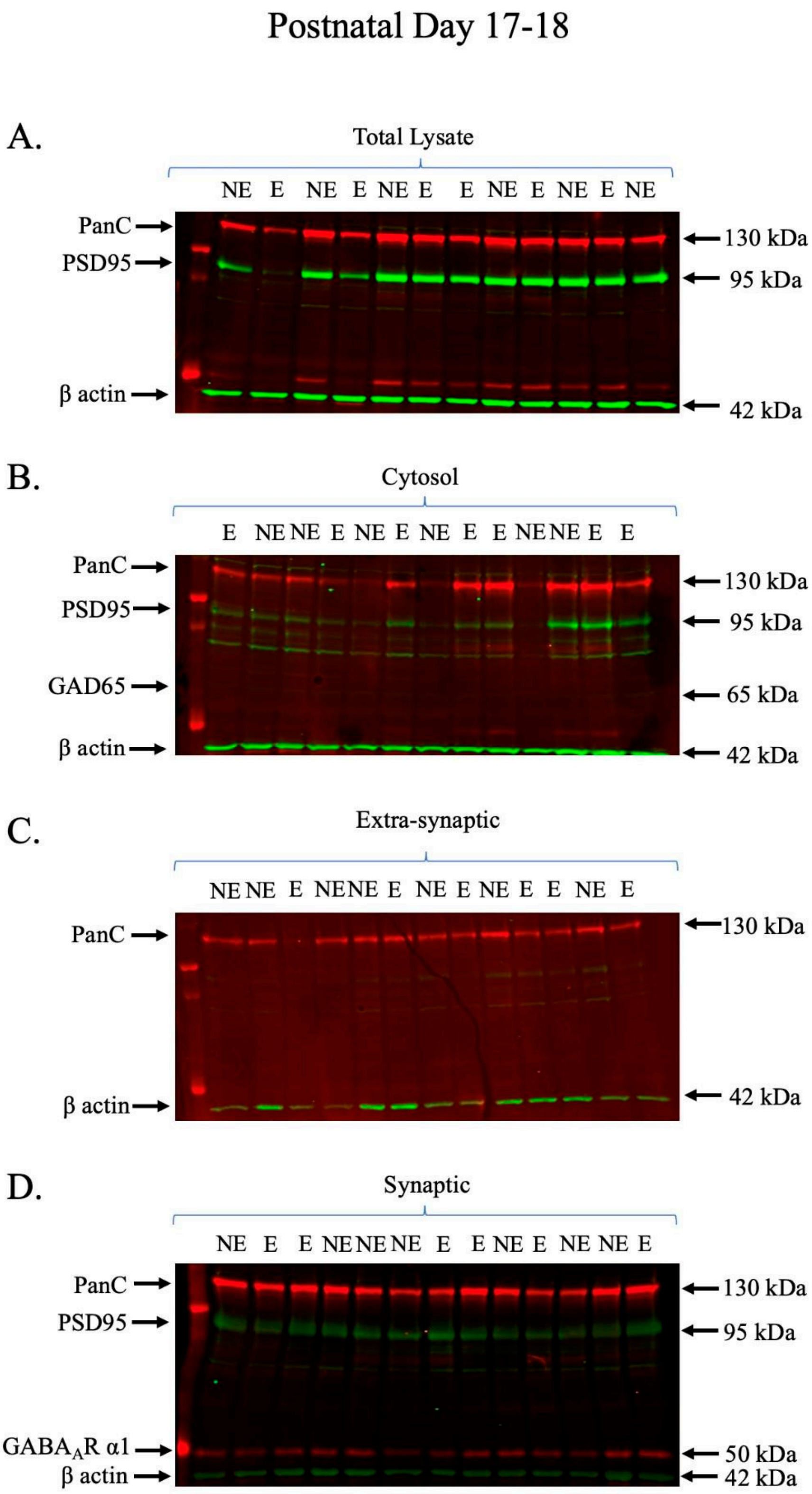


**Figure S3.** Representative western blots for subcellular fractions (total lysate, cytosol, extra-synaptic and synaptic) from primary somatosensory cortex of epileptic stargazers (E) and non-epileptic control littermates (NE) at postnatal days 17-18. (NE: Non-epileptic controls; E: epileptic stargazers).
